# How Is Learning Motivation Shaped Under Different Contexts: An Ethnographic Study in the Changes of Adult Learner’s Motivational Beliefs and Behaviors Within a Foreign Language Course

**DOI:** 10.3389/fpsyg.2018.01603

**Published:** 2018-08-30

**Authors:** Wenjin Vikki Bo, Mingchen Fu

**Affiliations:** ^1^Teaching & Learning Centre, Singapore University of Social Sciences, Singapore, Singapore; ^2^Institute of Moral Education, Nanjing Normal University, Nanjing, China

**Keywords:** motivational beliefs, motivational behaviors, foreign language learning, adult learners, classroom research

## Abstract

There has been a burgeoning interest of students’ motivational beliefs in determining their motivational behaviors in classroom activities: choice of task and persistence of task. Previous research mostly used quantitative methods to understand students’ general motivation, without taking contextual factors into consideration. To fill in this gap, the present study examined the influence of changing contexts on students’ motivational beliefs in a Chinese language classroom, and how those changes in motivational beliefs shaped their motivational behaviors in class activities. An ethnographic multiple-case study approach was adopted, and six adult learners were chosen from a Chinese language course in a Hong Kong university. On-going semi-structured interviews, class observations, stimulated recall and document reviews were conducted to understand student development across time. Findings show that the more proficient students were showing relatively stable motivational beliefs as well as behaviors throughout the foreign language course. In contrast, the less proficient students were demonstrating obvious changes in their motivational beliefs and hence behaviors, due to the different contexts of non-exam and high-stake exam. The study suggested students’ learning motivation in class was context-dependent, and could fluctuate substantially on a weekly basis. Those dynamic within-course changes at different learning stages and the reasons shaping the changes could give pedagogical insights to the teacher with adult learners.

## Introduction

It has been noted for long that although aptitude is a critical factor in language learning, motivation can actually go beyond those constraints to a large extent in language achievements (Gardner and Lambert, 1972). Extensive research in motivation has been conducted to examine how motivational beliefs influence students’ behaviors, namely choice and persistence in learning activities (Bong, 2001b; Durik et al., 2006; Denissen et al., 2007; Cole et al., 2008). To investigate this issue, longitudinal studies with younger students in K-12 schools have been conducted, attempting to track children’s and adolescents’ age-related changes from the current to the future (Wigfield et al., 1997; Wigfield and Eccles, 2000). Most of those studies indicated the change tendency of younger learners’ motivational beliefs on a semi-annual or annual basis, during elementary school and secondary school years; they also showed how those developmental beliefs ultimately influenced their behaviors on course level, such as choice of course (e.g., enrolment of the current course) and persistence of course (e.g., intention for future enrolment in the course) (Eccles et al., 1989; Wigfield et al., 1991, 1997).

The previous studies did provide a macro view of younger learners’ changing tendency across ages, but obvious limitations can be seen accordingly. First and foremost, the change of student’s motivational beliefs could take place in a much shorter duration than a yearly or semi-yearly basis, especially in a classroom setting (Dornyei, 2000). Second, motivational behaviors were not sufficiently examined, either, since self-report of questionnaire was the major or sole research instrument. Therefore, the major focus was on student’s motivational beliefs, lacking in the deep understanding of their motivational behaviors in a real-life context. Third, older students such as adult students have been under-researched, especially in the context of higher education. Considering the existing research gaps, suggestions for future studies of achievement motivation have been given – to evaluate the theories and previous findings with alternative research methods such as qualitative approaches, in contexts of other regions such as Asian countries, within different subjects such as languages other than English, and with different age groups such as adult learners (Hulleman et al., 2008; Wigfield and Cambria, 2010).

In the context of Hong Kong, Mandarin Chinese learning has been promoted rapidly during the last two decades, and has been used with a large population as a foreign language in work or social occasions in Hong Kong ever since. However, among the few previous studies of student motivation in Chinese learning in Hong Kong, they mainly used quantitative approaches (e.g., questionnaires) to investigate the subject of Cantonese Chinese with the age group of primary school students (Lau, 2009a) and secondary school students (Lau and Chan, 2001; Lau, 2009a,b). Those studies also just focused on understanding students’ general motivational orientations, without contextualizing the construct of motivation; furthermore, younger learners in K-12 education have been the targeted participants in most of the previous studies, and little research was found among the adult learners. Hence, the present study contributed to the research of learning motivation by using qualitative approach (n ethnographic study) to investigate the subject of Mandarin Chinese as foreign language in Hong Kong, with the age group of adult learners.

The theoretical framework for the present study is based on expectancy-value theory developed by Eccles et al. (1983) and her colleagues. In this framework, focus has been attached to how student’s motivational beliefs (ability beliefs and perception of task values) influence their motivational behaviors (choice and persistence of task) in the academic subjects, which matched the focus of the present study. To be specific, student’s ability beliefs refer to their own estimated competence to achieve success, while student’s perception of task values indicate the qualities of learning tasks and how those qualities attract students to do the task. Task values are classified into four types – intrinsic value (personal interest in the task/activity), attainment value (perceived importance to performance of the task/activity), utility value (usage of the task/activity) and cost value (anticipated time and effort in the task/activity).

Regarding how the motivational behaviors (choice and persistence) are determined by student’s motivational beliefs (ability beliefs and perception of task values), scholars have different opinions. On one hand, it is believed student’s ability beliefs are salient to determine their behaviors (Bandura, 1997; Graham and Williams, 2009; Schunk and Pajares, 2009). That is to say, if students believe they are able to succeed at learning tasks or activities, they will be more likely to choose them and persist in front of difficulties. On the other hand, it is argued student’s perception of task values is more influential to determine their behaviors, because if individuals see little value for doing a task or an activity, they would probably not choose it even if they might feel able to do it (Wigfield and Eccles, 1992; Wigfield et al., 2009).

Different from previous studies that mostly examined students’ motivational attitudes and behaviors from the subject-specific level, the present study was novel to explore the task-specific level in a classroom (of Chinese as foreign language, hereafter CFL). A Chinese (Mandarin) language class at intermediate level in a university in Hong Kong was chosen for the research, since the dropout rate of language classes at such a level was reported to be low, allowing for continuous data collection with sufficient students. In the investigated Chinese language class, there were 16 learners in total (5 males and 11 females), including 9 university students and 7 working professionals. The teacher who instructed the language class was a native speaker of Chinese Mandarin, named Li. She majored in Teaching Chinese as a foreign language, and had 2 years’ teaching experiences. Since the Chinese teacher Li was required to cover 6 units of the textbook (named “Contemporary Chinese 3,” published by the headquarter of Confucius Institute) for one semester’s course, the major consideration for Li to design the classroom tasks were based on the content of the textbook and the teaching approach is mostly lecturing. Accordingly, six types of tasks were observed in classroom activities: vocabulary, grammar, reading, listening, writing and speaking. Course assessments are made of mid-term exam and final exam, but only the pass of the final exam is the requirement to enter the next course. The study aimed at answering the two major research questions:

(1) How do adult learners’ motivational attitudes (ability beliefs and perception of task values) develop within a CFL course in the classroom setting?
(2) How do adult learners’ motivational behaviors (choice and persistence) develop within a CFL course in the classroom setting?

## Materials and Methods

This study is qualitative in nature, and is designed as an ethnographic study, adopting content analysis method. Considering the objectives of the study, ethnography is an effective methodology to understand participants’ social context, and to explore how they construct and interpret their beliefs as well as behaviors (Denzin and Lincoln, 2008). By adopting the ethnographic approach, the present study enabled us to examine how the different contexts in the classroom shaped the research participants’ motivation across time, including their motivational beliefs and motivational behaviors. It also allowed us to conduct an intensive and context-specific analysis among a comparatively small sample size (Gomm et al., 2000), like the current research with six participants. With the use of ethnographic case studies, it can effectively provide ‘a chronological narrative of events relevant to the cases’ with the focus on groups of individuals sharing similarities (Cohen et al., 2000). Hence, findings of the current study were presented in the chronological order from the beginning of the Chinese course to the end of it, including four learning stages; focus was on groups of students who had similar proficiency levels of Chinese language, and hence were observed to demonstrate similar patterns in their motivational beliefs and behaviors.

The researcher was participating all the sessions in class to understand student learning, and after class was also helping students with any questions they might have regarding the Chinese learning. This was useful for the researcher to build rapport with all the research participants, and understand their motivational changes across time within the course.

### Participants

Six learners were selected purposefully from a Chinese language course provided in a university language center in Hong Kong. All of them were in the intermediate-level language class, but with varying proficiency levels (see **Table 1**). Three were university students and three were working professionals (indicated as Professional in **Table 1**). A maximum variation sampling approach (Patton, 1990) was used, since each selected participant provided variation (nationality, occupation, Chinese proficiency level). This enabled the researcher to select information-rich cases to present a diverse range of perspectives, and identify patterns across multiple cases.

**Table 1:** Participant information.

Participant	Sex	Nationality	Age	Proficiency level in class	Occupation
Kelly	Female	United States	36	High	Professional
Amy	Female	United Kingdom	34	High	Professional
Andrew	Male	Italy	25	Average	Postgraduate
Sam	Male	Korea	21	Average	Undergraduate
Cora	Female	Switzerland	38	Low	Professional
Mike	Male	United States	28	Low	Postgraduate

### Data Collection

The major sources of data consisted of on-going individual interviews, class observations, and reflective discussions (stimulated recall) regarding the observational data with both the six participants and the teacher. Relevant documents (e.g., teaching plans, attendance records, etc.) were also collected as supplementary data. The integration of multiple sources across time enabled us to better understand the developments of students’ motivational beliefs, as well as their subsequent motivational behaviors in relation to the classroom contexts (Fulmer and Frijters, 2009). Interview questions semi-structured and were developed from the commonly used questionnaires of expectancy-value theory (Wigfield, 1994) with small modifications (**Appendix 1**). The observation scheme (**Appendix 2**) was developed from the component of ‘learners’ motivated behavior’ in MOLT Observation Scheme (Guilloteaux and Dörnyei, 2008), integrated with the definitions of students’ motivational behaviors in the expectancy-value theory (Wigfield and Eccles, 1992; Wigfield and Cambria, 2010).

The CFL course includes 20 sessions (18 were instruction sessions and 2 were exam sessions), lasting about 13 weeks. Class observations were conducted and audio-taped in all the 18 instruction sessions, followed by 18 rounds of stimulated recall with 6 participants (108 stimulated recall in total) and the teacher (18 stimulated recall in total) individually. Based on the observed developments of students’ behaviors in class, four rounds of individual interviews were carried out to understand the developments of their motivational beliefs. Stage 1 was the beginning of the semester (Session 1 to 4); Stage 2 was before the middle of the semester (Session 5 to Session 8); Session 9 was the mid-term test; Stage 3 was after the middle of the semester (Session 10 to Session 14); Stage 4 was around the end of the semester (Session 15 to 19); Session 20 was the final exam.

This study was carried out in accordance with the recommendations of Ethics Committee of University of Hong Kong with written informed consent from all subjects. The participants were students in a Chinese language course and enrolled in this study through their class instructor. Participation of the study was entirely voluntary and all participants gave written informed consent in accordance with the Declaration of Helsinki. The protocol was approved by Ethics Committee of University of Hong Kong.

### Data Analysis

Qualitative data from semi-structured interviews, observation field notes, and stimulated recall were analyzed inductively on principles established by Miles and Huberman (1994). The first author read the transcripts and then summarized each participant’s motivational trajectory within the course; this was triangulated and revised by other colleagues. Having analyzed the case of each participant, we followed the iterative procedure and conducted a cross-case analysis.

Descriptive quantitative data from class observations were analyzed primarily based on calculation of frequencies and percentages in each participant’s behaviors. As mentioned earlier, students’ motivational behaviors were examined and analyzed from the task-specific perspective in the present study, namely the choice of task, and persistence of task after encountering difficulties/mistakes. According to the observation, six types of tasks were observed in classroom activities: vocabulary, grammar, reading, listening, writing and speaking. The classification of six tasks in class were also triangulated with the instructor Li who designed them in the first place. According to Li, six different types of learning tasks were included in class based on the content of the textbook as well as the syllabus of the class.

As for the choice of task, the frequency for different types of tasks that each participant actively chose in class were counted and calculated into percentage. For example, if an individual was observed to have 10 active participations in total during a session, and 2 active participations were observed to be in listening task, his/her percentage in the choice of listening task would be 20%. Concerning the persistence of task, students’ instant reactions and subsequent active participation after making a mistake in class were recorded. In particular, the time interval between the mistake and the subsequent active participation was recorded for analysis. According to the findings, the time intervals were counted and categorized into ranges of 0 min (immediately), 0–3, 3–6, 6–9… 27–30 min, and more than 30 min. The categorization of the time intervals was data-driven. In other words, the exact time intervals of each participant were calculated and categorized into small time ranges that could also differ from one range to another. After counting the frequency of different time intervals, the researchers then assessed the percentage of each interval among individual participants. For example, if a participant made 5 mistakes in one particular session, and the time intervals between the mistakes and the subsequent active participations were once within the range of 0–3 min, once within the range of 3–6 min, and 3 times within the range of 6–9 min, the percentage for each interval during this session was 20%, 20 and 60%, respectively.

## Results

Four learning stages were observed from the data while tracking students’ motivational attitudes and behaviors throughout the CFL course. In general, the more proficient students (around and above average level of the class) were showing relatively stable beliefs and behaviors, while the less proficient students (below the average level) were demonstrating obvious changes due to their evolving perception of peer comparison and high-stake exam. The following paragraphs will present the findings in chronological order from Stage 1 to Stage 4. Each stage will first present participants’ motivational beliefs (including ability beliefs and task values), and then motivational behaviors (choice of task and persistence of task) in class, respectively.

### Stage 1 (S1): Absence of Peer Comparison

#### Motivational Beliefs: Vague Ability Beliefs; Utility Value for Practical Use

At the beginning of the course when students were not fully informed of each other’s proficiency levels, no social comparison was yet established among peers. Therefore, when asked about their own abilities in Chinese, all of the six participants showed optimistic beliefs, describing themselves as ‘around average level’ (Cora, S1), ‘trying to reach average’ (Mike, S1), ‘something like average’ (Andrew, S1) ‘around average’ (Sam, S1), ‘about average’ (Kelly, S1), ‘average’ (Amy, S1) in their individual interviews.

With focus on learning for communication purposes in workplace or daily life, the task value they perceived most in classroom activities was unanimously utility value at Stage 1. For example, Kelly, Amy and Cora valued vocabulary and grammar tasks most in class, because they all believe those two types of exercises could be directly useful to ‘produce more sentences’ (Kelly, S1), ‘speak out more’ (Amy, S1), and ‘have longer sentences’ (Cora, S1) while making a Chinese conversation with others. In addition to those two types of tasks, Andrew and Sam also considered listening as a critical task type to improve their communication skills, so their perceived utility value was in the three types of learning tasks in class: vocabulary, grammar and listening.

I want to focus on listening too... You need to understand what others are saying first, so you can respond accurately… So I think it’s useful to focus on improving my listening comprehension, then building my vocabulary as well as make sentences that are grammatically correct. (Andrew, S1)

I feel listening is also useful to focus on... If I can also understand people better, with the good knowledge of vocabulary and grammar, then I can finally communicate with them. I want to learn something useful that can help my communication in Chinese. (Sam, S1)

In comparison with the other five participants, Mike valued grammar tasks only, as he mentioned in his interview, ‘grammar is the fundamental part to connect all the vocabularies I know right now… so it is most useful for me to improve my Chinese while talking to people’ (Mike, S1).

#### Motivational Behaviors: Shaped by Utility Value

At the beginning of the course, all the six participants were observed to choose the learning tasks they considered useful, no matter they felt competent or not to perform those tasks. That is to say, even they might not be confident in certain tasks in class, they would still be willing to participate actively as long as they thought participation in those tasks could be useful for daily life or workplace. According to observational data (see **Appendix 3** for details), all of Kelly’s active participation in class was consistently in the tasks of vocabulary and grammar – the types of tasks she considered most useful, such as Session 1 (70% in grammar and 30% in vocabulary), Session 2 (62.5% in grammar and 37.5% in vocabulary), Session 3 (78% in vocabulary and 22% in grammar), and Session 4 (64% in grammar and 36% in vocabulary). Likewise, the same pattern was observed in Amy’s and Cora’s active participation (in vocabulary and grammar), Sam’s and Andrew’s active participation (in vocabulary, grammar and listening), as well as Mike’s (in grammar). Those tasks in which research participants were observed to be actively participating were all the type of tasks they perceived useful, regardless of their performance in class.

Similar to participants’ choice of task in class, their persistence was also mainly shaped by their perceived usage (utility value) of learning tasks at Stage 1. That is to say, in the absence of peer comparison, students did not consider mistakes in class as a bad thing, but instead considered as ‘useful learning opportunities to improve Chinese.’ Therefore, at this stage the six participants rarely showed negative reactions (such as avoiding the subsequent task or being silent) after failing the current task, but persisted in participating actively within short time. As can be seen from the time intervals between participants’ mistakes in class and their subsequent active participation (see **Appendix 4** for details), all the six participants were re-participating actively in class within 15 min after making mistakes, varying from 0 min (immediately) to 15 min (1/8 of each session’s time). Taking Kelly as an example, as can be seen in **Appendix 4**, the time intervals between her mistakes and subsequent active participations in class were mostly within 0–3 min/3–6 min (1 out of 2 times for each range, making up 50%, respectively) during Session 2, and 0–3 min (3 out of 4 times, making up 75%) during Session 3. As for Mike who kept making mistakes in all the 4 sessions, he still continued to actively participate in class soon after the mistake, mostly within 0–3 min during Session 1, within 9–12 min during Session 2 (2 out of 3 times, making up 67%) and Session 3 (all the 2 times), and 12–15 min during Session 3 (all the 4 times).

### Stage 2 (S2): Emergence of Peer Comparison

#### Motivational Beliefs: Ability Beliefs Established in Hierarchical Orders; Attainment Value in Less Proficient Participants; Utility Value in More Proficient Participants

After the first several sessions, students were increasingly aware of classmates’ proficiency levels and started to compare with each other. As a result, all the six participants’ ability beliefs were clearly established in hierarchical levels: at low level (cases of Cora and Mike), at medium level (cases of Andrew and Sam), and at high level (cases of Kelly and Amy). With the disparity in the level of ability beliefs, participants’ perception of task values started to differ accordingly at this stage.

The less proficient participants (Cora and Mike) whose ability beliefs gradually decreased to a low level were shifting their focus from useful learning to classroom performance, indicating their attainment value in class activities at Stage 2. The following extracts suggest that participants with obvious decrease in ability beliefs tended to value certain tasks that could demonstrate their abilities and conceal their inabilities in class.

Initially I thought I was at the average level in this class, but now as you probably noticed I had to be the weakest link in this class! I really hate listening tasks because I had no idea what they were talking about… Vocabulary task was easier, so I like it much more in class. I hope we can do more vocabulary… (Cora, S2)

The same shift in task values was identified in Mike, who used to value grammar task at the beginning because of its usefulness. After realizing he was incompetent to perform grammar task in class, Mike started to value less grammar task, and value more vocabulary task – the type of task he felt more competent to perform in class activities.

You have definitely recognized that I’m one of the weakest students in class now… Grammar was too hard, and listening was just impossible for me, so I was definitely trying to avoid listening as much as possible… Vocabulary was the only type I felt I could get by in class so I like it more… (Mike, S2)

In contrast, for the remaining four participants, this focus on classroom performance was only observed slightly in participants with medium ability beliefs (Andrew and Sam), or none in participants with high ability beliefs (Kelly and Amy). When asked about which types of tasks they valued most in class, the major consideration was still the ‘usage’ and ‘usefulness,’ indicating the utility value. For example, although Andrew and Sam started to realize the proficiency gap between them and other more proficient students, the emphasis on useful learning still prevailed over the classroom performance.

Everybody wants to show people you know something, and you will feel good, but I don’t mind making mistakes, either, as long as I can learn something useful… My ultimate objectives in mind will be something like traveling to China… and be able to use Chinese there… (Andrew, S2)

Good performance seems important because they give you confidence, but mistakes can be useful to improve as well. I’m here to learn as much as possible and eventually be able to use Chinese with people in real life. (Sam, S2)

#### Motivational Behaviors: Less Proficient Participants’ Shaped by Ability Beliefs; More Proficient Participants’ Shaped by Utility Value

With the emerging peer comparison among students at Stage 2, ability beliefs seemed to shape the active participation of the less proficient students. According to the percentage of active participation, Cora and Mike were observed to mainly choose the types of tasks they felt competent to perform (vocabulary), and avoid the types of tasks they felt incompetent in class (listening). To specify, as shown in **Appendix 5**, the proportions of both Cora’s and Mike’s active participation in vocabulary suddenly became the highest at this stage in all the sessions, being 67% (Cora) and 67% (Mike) during Session 5, 67% (Cora) and 70% (Mike) during Session 6, 62.5% (Cora) and 50% (Mike) during Session 7, 75% (Cora) and 80% (Mike) during Session 8. They explained those behaviors as ‘a way to feel more confident’ (Cora, S2) and ‘a strategy to avoid mistakes’ (Mike, S2) in the stimulated recall. With the decreasing ability beliefs at Stage 2, Cora and Mike were showing weaker persistence to avoid challenges and conceal potential inabilities. The observed time intervals between their mistakes and the subsequent active participation were getting much longer compared to the beginning of the course. As shown in **Appendix 6**, this phenomenon was especially evident for Mike whose ability beliefs were tremendously dropping at Stage 2, and hence was constantly observed to keep inactive for more than 20 min (such as Session 7) or even 30 min (such as Session 5) after making a mistake. Notably, temporary strong persistence was observed in particular sessions (Session 6 and 8) when they made fewer mistakes in class and hence felt temporarily more competent.

In comparison, for the students whose ability beliefs were relatively high – either at medium (Andrew and Sam) or high level (Amy and Kelly), their choice of tasks didn’t change much at Stage 2, but remained the same as Stage 1. They were still observed to actively participate in the tasks they considered useful, despite the constant mistakes they made while performing the tasks, such as listening task for Andrew (being his second most active participation in Session 5 and Session 7 when listening tasks were included in class), grammar task for Sam (being his Top 2 active participations in all the sessions) as well as Amy (being her Top 2 active participations in all the sessions), and vocabulary task for Kelly (being her Top 2 active participations in all the sessions). In order to get as much useful skills as possible in class, they were maintaining the strong persistence as the previous stage, and were observed to persist in the subsequent task soon (mostly within 6 min) after failing the current task (see details in **Appendix 6**).

### Stage 3 (S3): Reinforcement of Peer Comparison

#### Motivational Beliefs: Ability Beliefs Changed by Mid-Term Test Scores; Attainment Value in Less Proficient Participants; Utility Value in More Proficient Participants

After the mid-term test in Session 9, participants’ awareness of peer comparison was getting stronger at this stage. As soon as they received the results of mid-term test, their ability beliefs underwent immediate changes according to the level of the scores. For the more proficient participants who received top scores (such as Kelly, Amy, Andrew and Sam), their ability beliefs were maintained at a high level. For the less proficient participants who received bottom scores (Cora and Mike), their ability beliefs were drastically declining accordingly.

With the ‘embarrassing score’ of the mid-term test, as described by both Cora and Mike in individual interviews at Stage 3, they felt the need to ‘pick up confidence from other sources’, and classroom activity was the major source. Therefore, the focus on classroom performance was even getting stronger for Cora and Mike at this stage, demonstrating their attainment value in classroom activities.

When you are feeling so lost in the exam, you need something else to let you know you are on the right track. I have to prove myself in the class activities… I hope we can do more writing in class because I am better in writing… (Cora, S3)

I got thirty something in the mid-term test, and that was humiliating. I almost got nothing correct in listening and grammar. I wish I could do better in class activities after the big failure in the mid-term test, but it was just impossible because we are doing lots of listening and grammar exercises, which were my weak parts… (Mike, S3)

Different from Cora and Mike, the remaining four participants received top scores from the mid-term test, resulting in an increase of confidence and sense of achievement.

I was expecting myself to do worse in listening… so I’m happy I just made 4 mistakes out of all the listening, and this exam was taken from HSK Level 4, which is above our class level, so it gave ma huge confidence boost… For the written part, it was easy and I did quite well, too… (Andrew, S3)

I think I could have improved my grammar and maybe vocabulary as well, but in general, I was quite happy about the mid-term exam… It was better than expected. (Sam, S3)

As far as I know, I got the highest scores in class… I think I have just more experience than other people so it is normal… It is just not fair they should be competing with me… I’m always good in exams anyway… (Kelly, S3)

The teacher told me my score is the second highest in class, so I’m satisfied. I think I could have done better with listening though… Anyway, I’m happy with the recognition. (Amy, S3)

With the ability beliefs at high level, their focus remained the same as previous stages – ‘learning useful skills’ for communication purpose, such as for traveling (Sam), career plans (Sam and Andrew), or daily use with Chinese friends (Kelly and Amy). Hence, the potential usage, namely utility value of the task was their major consideration in Chinese learning.

#### Motivational Behaviors: Less Proficient Participants’ Shaped by Ability Beliefs; More Proficient Participants’ Shaped by Utility Value

With the strengthened peer comparison after the mid-term test, ability beliefs continued to influence the active participation of less proficient students (Cora and Mike), who were observed to selectively choose the tasks they felt competent to perform (vocabulary), and avoid the tasks they felt incompetent (listening). According to observational data, the major part of both Cora’s and Mike’s active participation in class was in vocabulary – the same type of tasks they felt ‘relatively more confident to do (Cora, S3), or simply ‘easier to perform well because of the dictionary at hand’ (Mike, S3). To exemplify, as elaborated in **Appendix 7**, Cora’s most active participation in class was constantly in the type of vocabulary tasks, accounting for 54, 64, 67, 83, and 80% of her overall active participation during Session 10 to Session 14, respectively. Similarly, during the sessions Mike attended, his most active participation in class was also in the type of vocabulary tasks, accounting for 58, 100, and 100% of his overall active participation during Session 11, 12, and 13. With the continuously dropping ability beliefs, Cora and Mike were generally demonstrating weak persistence in class, since they often showed long-lasting silence (more than 30 min, which was more than ¼ of the session time), after making mistakes in class in order to avoid challenges and conceal inabilities in front of peers. Notably, temporary strong persistence with much shorter time intervals (3–6 min or 6–9 min) was observed in Session 10 and 11 (see **Appendix 8** for details) when there were only three students present in class, and hence ‘less peer comparison was sensed’ (Cora, S3), resulting in ‘less concern to avoid challenges’ (Mike, S3) among the less proficient students.

On the other hand, the more proficient students who held high ability beliefs still maintained their focus on learning Chinese for practical use (utility value) – the same as Stage 1 and 2 previously. Therefore, they were more likely to choose the tasks they considered useful, although they might make constant mistakes while performing the task (e.g., vocabulary task in particular). Strong persistence was consistently demonstrated in all the sessions at this stage, since they were observed to persist in the subsequent task shortly (all within 0–9 min) after failing the current task.

### Stage 4 (S4): Context of High-Stake Exam

#### Motivational Beliefs: Ability Beliefs Remained the Same as Stage 3; Utility Value (for Exam Preparation) in Less Proficient Students; Utility Value (for Practical Use) in More Proficient Students

With the final exam approaching, the focus of less proficient students was shifting from classroom performance to skills development, although for the sake of final exam – which was described as ‘an official summary of this language course’ (Mike, S4) and ‘the requirement to continue the next level’s course’ (Cora, S4). Therefore, their major consideration in classroom was how useful the learning tasks could help them acquire sufficient skills to pass or excel in the exam, indicating the utility value for the exam preparation.

My goal is that I want to do this exam at the maximum level I can do… The exam is absolutely influential and the score is absolutely important, so I hope the teacher can design more activities in class that are useful for the exam… (Cora, S4)

As you know, I basically failed the mid-term exam, so I really hope to do better in the final exam… That’s my current goal for Chinese learning. It will be great if the teacher can do some review for us in class so we will not be so lost in the exam later… (Mike, S4)

Compared to Cora who wanted to outstand in the final exam, Mike said in the interview that he ‘simply wanted to pass the exam,’ so he could have ‘a fair conclusion for this course.’

In contrast, the remaining four participants (Andrew, Sam, Kelly, and Amy) whose proficiency levels were relatively higher in class, did not perceive the fear to fail or the need to achieve in the final exam. Therefore, their focus in classroom was not much influenced by the approaching exam, but still remained the same as the previous stages – to use the skills gained from classroom (utility value) for communication in workplace or daily life.

The final exam is not the final goal for me. Whether I can use what I learnt in classroom back to real life is more important. (Andrew, S4)

Exam might be important, but being able to talk to people in Chinese is more important, with my friends or colleagues. Otherwise, it’s no use. (Sam, S4)

I really want to use Chinese more in my life, so apart from the exam, the ultimate goal here is to be able to use Chinese with other people. (Amy, S4)

I have been learning Chinese for long, but still feel there is a lot of things I need to know, a lot of vocabularies to learn, in order to communicate in Chinese. That’s the major purpose here. (Kelly, S4)

### Motivational Behaviors: Shaped by Utility Value

Unlike Stage 2 and 3, when Cora and Mike emphasized on good performance in the class activities and avoiding making mistakes, their focus was shifting to the good performance in the final exam at Stage 4. That is to say, as long as certain tasks in the class activities were useful to gain the skills for the final exam, they would actively choose to participate, including those tasks in which they felt incompetent. Based on observational data (**Appendix 9**), most of Cora’s and Mike’s active participation at this stage was not only in vocabulary – the type of tasks in which they felt relatively more competent, but also in grammar and listening – the two types of tasks in which they had always been feeling incompetent and had tried to avoid during the previous stage. In particular, for the very first time in this course, listening became the most active participation of Cora’s in Session 15 (accounting for 40% of her overall active participation), and of Mike’s in both Session 15 (accounting for 50% of his overall active participation) and Session 17 (accounting for 60% of his overall active participation). They explained this change of their behaviors as follows in their stimulated recall:

The priority is the final exam now, and listening exercise is really useful for the exam. (Cora, S4)

As long as I don’t make many mistakes in the exam, I don’t mind making them now in the class. (Mike, S4)

In order to learn as much as possible from the class activities and get ready for the final exam, they were also demonstrating strong persistence even they kept failing the tasks in class. No avoidance behaviors (such as long-lasting silence after making mistakes) were observed at this stage, but instead they were both observed to persist with subsequent active participations within short time intervals, mostly within 3–9 min for Cora, and 6–12 min for Mike (see details in **Appendix 10**).

On the other hand, the more proficient students whose ability beliefs were relatively higher were not much influenced by the final exam, but instead maintained the same focus as all the previous stages – mastering useful language skills for communication. Strong persistence was still demonstrated, since all of them were observed to persist with active participations within 0–6 min after making a mistake in class.

As shown in findings of Stage 1 to 4, more proficient students (including average and strong students in class) held relatively high ability beliefs throughout the CFL course. Therefore, their motivational behaviors were relatively stable in class activities, constantly shaped by their perception of utility value in the tasks. In contrast, less proficient students (weak students) demonstrated obvious changes in their ability beliefs, so their achievement behaviors varied accordingly from stage to stage, depending on specific contexts in class.

## Discussion

In this part I raise a cross-case discussion based on the findings of the six participants, and attempt to relate the present study to the literature review, regarding participants’ motivational beliefs as well as motivational behaviors in the CFL classroom. As can be seen in the previous section of findings, the more proficient students did not demonstrate obvious changes in motivation, whereas the less proficient students’ motivation in classroom was continuously changing due to two major contextual factors: (1) peer comparison leading to the weakened ability beliefs, and hence the decrease of motivation (non-exam context) and (2) exam preparation leading to the strengthened task values, and hence the increase of motivation (high-stake exam context).

### Classroom Motivation in Non-exam Context

In non-exam context, peer comparison seemed to be the trigger of students’ motivational change. When peer comparison is not yet established among students, they usually tend to hold optimistic ability beliefs, and focused on mastering skills for practical use outside class. Therefore, with the focus on utility value in learning tasks, students would be more likely to choose the tasks they considered useful in class, no matter they felt competent or not to perform those tasks. With the strong motivation to participate in class, they would also persist more even in front of challenges and mistakes, for the sake of more useful learning. This corroborates prior research to some extent that students’ focus on skills development was often correlated with utility value and intrinsic value (Bong, 2001a; Xiang et al., 2004), although only utility value was evidently shown in the present study. The likely explanation is that most of the previous research was investigating younger students in primary schools who are more likely to perceive intrinsic value than adult students in the current research.

As a matter of fact, the evolving perception of task values from younger students to older students has been pointed out earlier by scholars in motivation research (Anderman and Maehr, 1994; Lepper et al., 2005) – as younger students grew older and entered further grades during K-12 education, their interest (intrinsic value) in learning would gradually drop, but the perceived usage (utility value) in learning tasks became increasingly significant, especially when students’ long-term goals were gradually established. The present study sheds light on the further trajectory that when students finish K-12 education and enter higher education, the intrinsic value might even be gradually replaced by utility value in learning tasks.

Notably, when the students are increasingly aware of classmates’ proficient levels after several sessions, the less proficient students would perceive a threat to their ability beliefs, and started to focus more on demonstrating their abilities. According to Jacobs and Eccles (2000), it was a natural reaction when students’ ability beliefs were weakened through peer comparison in the ‘pecking order’ of a class. Therefore, the less proficient students would selectively choose the tasks they felt competent to demonstrate their abilities and avoid the tasks they felt incompetent to conceal their inabilities. With the decreasing motivation, they would easily give up trying when encountered difficulties, demonstrating weak persistence.

The influence of declining ability beliefs upon the less proficient participants’ task values was in accordance with Bandura (1999) who maintained ability belief is usually the prior causal factor of perceived values in learning tasks. This was also the context when ability beliefs were more influential to students’ motivational behaviors compared to task values. In another word, even when students perceive values in the task, they would not choose to participate or persist in trying if their ability beliefs are undermined (Bandura, 1997). This phenomenon would become more obvious when the less proficient students realized the further proficiency gap between them and the rest of the class, such as in a mid-term test of the present study. This observed phenomenon develops the theoretical proposition with empirical data that students who doubt their abilities, especially under the influence of bad test scores, will take actions to protect their identity, usually by avoiding tasks in class that might reveal their incompetence (Urdan and Schoenfelder, 2006). Our findings suggest peer comparison is the reason behind this pattern in non-exam context.

### Classroom Motivation in the High-Stake Exam Context

Based on students’ motivational development within the course, high-stake exam, however, seemed to exert a stronger impact compared to peer comparison. This is because the less proficient students’ focus on ability demonstration gradually shifted to useful skills (utility value) as the final exam approached, although for the sake of the exam result – which would determine their eligibility for the next level’s course. This change due to the high-stake exam exemplifies the previous literature that exams could usually adjust students’ perception of task values and promote their learning motivation (Wise and DeMars, 2005; Cole et al., 2008), but the present study argues the impact is limited to the less proficient students who feel ‘the fear to fail or need to achieve’ (Elliot and McGregor, 2001). Therefore, even the less proficient students still sense the peer comparison and still hold low ability beliefs before the exam, their motivation to participate and persist in class would become stronger during the exam period. It is worth mentioning that while high-stake exam has been recognized as a critical factor in the enhancement of student motivation (Hong and Peng, 2008; Eklöf, 2010), our findings suggest that the context of highs-stake exam is not as influential to the more proficient students who tend to demonstrate stable and high motivation both in the exam and non-exam contexts.

### Theoretical Implications

Building on previous research of expectancy-value theory and the findings of the present study, to better conceptualize the dynamic construct of learning motivation, we suggest more discussion is needed on (1) the age of learners, (2) the proficiency levels of learners, and (3) the contexts of learning. To begin with, the age of learners play a significant role in their motivational beliefs: while younger learners were found to focus on both the usefulness (utility value) and interest (intrinsic value) of learning tasks (Eccles and Wigfield, 2002; Linnenbrink, 2005), our study suggest that adult learners tend to prioritize the usefulness over the interest of the learning tasks. Hence, they would tend to participate more in those tasks they considered useful. This could also provide insights to front-line teachers while designing learning tasks in class.

Furthermore, learning motivation is highly dynamic (Hotho, 2000) and our findings further explain the motivational development is largely dependent on learners’ proficiency levels. On one hand, the perceived values of learning tasks among the more proficienct students are usually more stable, since their ability beliefs don’t fluctuate as much as the less proficient students. With the relatively stable motivaitonal beliefs, their motivational behaviors (e.g., active participation in learning activities) would often maintain quite steady as well. In comparison, when the less proficient students sense a threat to their ability beliefs, they would mostly value the tasks that could either demonstrate their abilities or conceal their inabilities (Bandura, 1999; Urdan and Schoenfelder, 2006). As a result, their motivational behaviors would change accordingly: they would be more likely to actively participate in the tasks they feel competent in.

Last but not least, our study suggests that motivational change in the less proficient students will also vary in different learning contexts, which was insufficiently explored in the previous research of expectancy-value theory. As indicated by the findings, the less proficient students’ learning motivation was shaped differently in the non-exam context from the high-stake exam context. We argue that in the high-stake exam context, exam preparation seems to exert a stronger impact than peer comparison on the less proficient students’ motivation; therefore, although those students could still sense the threat to their ability beliefs, they would eventually choose to participate in the useful tasks that might even reveal their inabilities in front of peers. While the context of high-stake exam has been recognized to promote students’ motivational beliefs and behaviors (Wise and Kong, 2005; Eklöf, 2010), the prestent study argues such a context appears to only shape the learning motivation of the less proficient students.

## Conclusion

The present study was novel to investigate students’ motivational changes on the task-specific basis in the classroom setting, while most of previous studies focused on the change at the subject-specific level. In particular, the observed developments in participants’ motivation within the CFL course contributed to understanding contextual factors in the construct of motivation – a dynamic and context-dependent construct, which could fluctuate from day to day or week to week, as a result of a specific test or a particular lesson (Hotho, 2000). While tracking students’ motivational development within a foreign language course, this study suggested the important distinction between the more proficient students and less proficient students – the former tend to hold stable and high motivation in class while the latter are likely to demonstrate continuous fluctuations due to the contextual changes. In the non-exam context, the emergence of peer comparison was discovered to be the reason to shape motivational change: from focus on developing useful skills to focus on demonstrating good performance. However, the impact of peer comparison seemed to fade in the context of high-stake exam, and students’ learning focus shifted to developing useful skills again, although for the sake of exam preparation. The present study argues that although significant progress has been made to explore the contextual factors in motivation, current research paradigms are still lacking in explaining the dynamic motivational fluctuations within a shorter duration, such as a complete course, a learning stage, or an exam period. Those understandings, however, are essential for pedagogical practice in the classroom setting. The research reported here has attempted to shed some light on issues of adult learners’ motivational development on weekly basis throughout the entire foreign language course, which could give direct insights to language teachers and help to promote student motivation in classroom.

The present study is small-scale in nature, and hence is limited to make generalizations to a larger population (Donmoyer, 1990). However, as Yin (2009, pp. 38–39) mentioned, the findings of small-scale qualitative research could provide insights to studies with similar contexts, and achieve “analytical generalization” in contrast to “statistical generalization.” Future studies could attempt to explore the motivational changes among a larger sample in a CFL classroom, especially in CFL courses at different levels. For example, the present study was conducted in a CFL course at intermediate level, so future research could explore the language classes at other levels, such as entry level or advanced level, to see whether any differences exist. Furthermore, this study focused on students’ motivational beliefs and behaviors in classroom activities while learning Chinese as a foreign language. In order to fully understand their motivation, it is important for future studies to examine students’ motivational beliefs and behaviors in after-school activities, and understand the reasons causing the difference, if any.

## Author Contributions

WB conducted the study and wrote up the first draft of the manuscript. MF participated in the designing of the study and helped revise the manuscript.

### Conflict of Interest Statement

The authors declare that the research was conducted in the absence of any commercial or financial relationships that could be construed as a potential conflict of interest.

## Appendix

**APPENDIX 1:** Interview questions.

(1) In general, do you find the classroom tasks interesting at this stage?
(2) Which type(s) of tasks is/are most interesting to you? Which type(s) is/are least interesting?
(3) How important is it for you to be good at the classroom tasks at this stage?
(4) In general, do you find the classroom tasks useful to you at this stage?
(5) Which type(s) of tasks is/are most useful to you? Which type(s) is/are least useful? Why?
(6) Compared to other students, how well do you expect to do in the classroom tasks at this stage?
(7) Did your expectancies to perform well change (increase/decrease) compared to the previous stage? If yes, how?
(8) How competent do you feel with the classroom tasks at this stage?
(9) Which types of tasks do you feel most competent in class activities? Which types of tasks do you feel least competent in class activities?

**APPENDIX 2:** Observation scheme of students’ achievement behaviors in classroom activities.

Achievement behaviors	Time (minutes)/Aspects to examine	1	2	3	4	5
Choice of tasks	Voluntary answers To the teacher/classmates					
	Initiated questions					
	Active discussions with the teacher/classmates					

Performance of tasks	Correct answers					
	Incorrect answers					

Persistence of tasks	Time intervals between the mistake and subsequent active participation					
	Instant reactions after mistakes (if any)					

**APPENDIX 3:** Choice of task in class at Stage 1: percentage (and frequency) of active participation.

	Kelly	Amy	Andrew	Sam	Cora	Mike
Session 1: Top 1 choice	Grammar 70% (7)	Vocabulary 50% (1)	Vocabulary 64% (11)	Vocabulary 38% (6)	Grammar 67% (2)	Grammar 100% (1)
Session 1: Top 2 choice	Vocabulary 30% (3)	Grammar 50% (1)	Grammar/Listening 18% (3)/18% (3)	Listening 25% (4)	Vocabulary 33% (1)	None
Session 2: Top 1 choice	Grammar 62.5% (5)	Grammar 50% (2)	Vocabulary 56% (9)	Listening 50% (4)	Grammar 83% (5)	Grammar 100% (4)
Session 2: Top 2 choice	Vocabulary 37.5% (3)	Vocabulary/Reading 25% (1)	Grammar 25% (4)	Vocabulary/Grammar 25% (2)	Vocabulary 17% (1)	None
Session 3: Top 1 choice	Vocabulary 78% (7)	Vocabulary 100% (2)	Vocabulary 60% (12)	Vocabulary 50% (8)	Grammar 60% (3)	Grammar 100% (4)
Session 3: Top 2 choice	Grammar 22% (2)	None	Grammar/Listening 20% (4)	Grammar 44% (7)	Vocabulary/Listening 20% (1)	None
Session 4: Top 1 choice	Grammar 64% (9)	Vocabulary 50% (1)	Vocabulary 65% (15)	Vocabulary 80% (8)	Vocabulary 50% (3)	Grammar 83% (5)
Session 4: Top 2 choice	Vocabulary 36% (5)	Grammar 50% (1)	Grammar 35% (8)	Grammar 20% (2)	Grammar 50% (3)	Vocabulary 17% (1)

*In Session 4, no listening tasks were included.*

**APPENDIX 4:** Persistence of task in class at Stage 1: percentage (and frequency) of time intervals (between mistake and subsequent active participation) (time interval in minutes).

	Kelly	Amy	Andrew	Sam	Cora	Mike
Session 1: Top 1 frequent time interval	None	None	0–3 67% (2)	3–6 100% (1)	None	0–3 100% (1)
Session 1: Top 2 frequent time interval	None	None	0 33% (1)	None	None	None
Session 2: Top 1 frequent time interval	0–3/3–6 50% (1)/50% (1)	6–9 100% (1)	None	None	6–9 100% (1)	9–12 67% (2)
Session 2: Top 2 frequent time interval	None	None	None	None	None	0–3 33% (1)
Session 3: Top 1 frequent time interval	0–3 75% (3)	0–3 100% (1)	3–6 67% (2)	0–3 50% (3)	6–9 67% (2)	9–12 100% (2)
Session 3: Top 2 frequent time interval	0 25% (1)	None	6–9 33% (1)	0/6–9/12–15 17% (1)/17% (1)/17% (1)	9–12 33% (1)	None
Session 4: Top 1 frequent time interval	None	None	0–3 100% (1)	3–6 100% (1)	6–9 100% (1)	12-15 100% (4)
Session 4: Top 2 frequent time interval	None	None	None	None	None	None

**APPENDIX 5:** Choice of task in class at Stage 2: percentage (and Frequency) of active participation.

	Kelly	Amy	Andrew	Sam	Cora	Mike
Session 5: Top 1 choice	Vocabulary 53%(8)	Grammar 50%(3)	Vocabulary 54%(7)	Listening/Vocabulary 37.5% (3)/37.5% (3)	Vocabulary 67%(4)	Vocabulary 67%(2)
Session 5: Top 2 choice	Grammar 40%(6)	Vocabulary 33%(2)	Grammar/Listening 23%(3)/23% (3)	Grammar 25%(2)	Grammar/Writing 17%(1)/17% (1)	Grammar 33%(1)
Session 6: Top 1 choice	Vocabulary 68%(13)	Vocabulary 62.5% (5)	Vocabulary 54%(7)	Grammar 50%(4)	Vocabulary 67%(4)	Vocabulary 70%(7)
Session 6: Top 2 choice	Grammar 32%(6)	Grammar 37.5% (3)	Grammar 31%(4)	Vocabulary 37.5% (3)	Writing 33%(2)	Grammar 30%(3)
Session 7: Top 1 choice	Vocabulary 53%(8)	Vocabulary 60%(3)	Vocabulary 67%(18)	Listening 42%(5)	Vocabulary 62.5% (5)	Vocabulary/Listening 50%(1)/50% (1)
Session 7: Top 2 choice	Grammar 33%(5)	Grammar 40%(2)	Grammar/Listening 15%(4)/15% (4)	Vocabulary/Grammar 25%(3)/25%(3)	Grammar 25%(2)	None
Session 8: Top 1 choice	Grammar 54%(14)	Vocabulary 60%(3)	Vocabulary 63%(27)	Vocabulary 66%(4)	Vocabulary 75%(6)	Vocabulary 80%(4)
Session 8: Top 2 choice	Vocabulary 35%(9)	Grammar 40%(2)	Grammar 35%(15)	Grammar/Speaking 17%(1)/17% (1)	Grammar 25%(2)	Grammar 20%(1)

*In Session 6 and Session 8, no listening tasks were included; In session 7, Mike checked the correct answers before all her active participation.*

**APPENDIX 6:** Persistence of task in class at Stage 2: percentage (and Frequency) of time intervals (between mistake and subsequent active participation) (time interval in minutes).

	Kelly	Amy	Andrew	Sam	Cora	Mike
Session 5: Top 1 frequent time interval	3–6 50% (2)	3–6 100% (2)	0 100% (2)	0/0–3 50% (1)/50% (1)	15–18 50% (3)	>30 60% (6)
Session 5: Top 2 frequent time interval	0–3/6–9 25% (1)/25% (1)	None	None	None	12–15 33% (2)	21–23 20% (2)
Session 6: Top 1 frequent time interval	0–3 67% (2)	None	None	0 75% (3)	6–9 100% (1)	3–6 100% (2)
Session 6: Top 2 frequent time interval	3–6 33% (1)	None	None	3–6 25% (1)	None	None
Session 7: Top 1 frequent time interval	0–3 100% (1)	None	0–3 100% (3)	0–3 100% (3)	None	21–23/23–25 50% (2)/50% (2)
Session 7: Top 2 frequent time interval	None	None	None	None	None	None
Session 8: Top 1 frequent time interval	None	3–6/9–12 50% (1)/50% (1)	0 62.5% (5)	None	6–9 100% (2)	6–9 100% (2)
Session 8: Top 2 frequent time interval	None	None	3–6 37.5% (3)	None	None	None

**APPENDIX 7:** Choice of task in class at Stage 3: percentage (and Frequency) of active participation.

	Kelly	Amy	Andrew	Sam	Cora	Mike
Session 10: Top 1 choice	Absent	Vocabulary 60% (3)	Vocabulary 73% (19)	Absent	Vocabulary 54% (7)	Absent
Session 10: Top 2 choice	Absent	Listening 40% (2)	Listening 19% (5)	Absent	Listening 38% (5)	Absent
Session 11: Top 1 choice	Absent	Absent	Vocabulary 74% (20)	Absent	Vocabulary 64% (7)	Vocabulary 58% (7)
Session 11: Top 2 choice	Absent	Absent	Grammar 26% (7)	Absent	Grammar 27% (3)	Grammar 42% (5)
Session 12: Top 1 choice	Vocabulary 43% (6)	Vocabulary 62.5% (5)	Vocabulary 76% (26)	Vocabulary 76% (22)	Vocabulary 67% (6)	Vocabulary 100% (1)
Session 12: Top 2 choice	Grammar 36% (5)	Grammar 37.5% (3)	Grammar 15% (5)	Grammar 14% (4)	Writing 22% (2)	None
Session 13: Top 1 choice	Vocabulary 56% (10)	Vocabulary 100% (2)	Vocabulary 74% (17)	Vocabulary 50% (11)	Vocabulary 83% (5)	Vocabulary 100% (1)
Session 13: Top 2 choice	Listening 33% (6)	None	Grammar 22% (5)	Listening 27% (6)	Listening 17% (1)	None
Session 14: Top 1 choice	Vocabulary 100% (3)	Vocabulary 100% (2)	Vocabulary 75% (15)	Vocabulary 100% (7)	Vocabulary 80% (4)	None
Session 14: Top 2 choice	None	None	Grammar 15% (3)	None	Grammar 20% (1)	None

*In Session 10 and 11, only three students were attending the class; In Session 11, 12 and 14, no listening tasks were included; In Session 13, Cora checked the right answers before her active participation in listening tasks.*

**APPENDIX 8:** Persistence of task in class at Stage 3: percentage (and Frequency) of time intervals (between mistake and subsequent active participation) (time interval in minutes).

	Kelly	Amy	Andrew	Sam	Cora	Mike
Session 10: Top 1 frequent time interval	Absent	None	0–3 67% (4)	Absent	3–6 56% (5)	Absent
Session 10: Top 2 frequent time interval	Absent	None	0 33% (2)	Absent	6–9 44% (4)	Absent
Session 11: Top 1 frequent time interval	Absent	Absent	0–3 100% (3)	Absent	3–6 100% (2)	3–6 100% (3)
Session 11: Top 2 frequent time interval	Absent	Absent	None	Absent	None	None
Session 12: Top 1 frequent time interval	6–9 100% (2)	None	0–3 100% (3)	0–3 50% (2)	21–23 100% (2)	>30 67% (4)
Session 12: Top 2 frequent time interval	None	None	None	0/3–6 50% (2)/50% (2)	None	28–30 33% (2)
Session 13: Top 1 frequent time interval	0–3 100% (3)	6–9 100% (2)	0–3 86% (6)	0–3 67% (2)	28–30 50% (3)	>30 50% (4)
Session 13: Top 2 frequent time interval	None	None	0 14% (1)	3–6 33% (1)	>30 33% (2)	25–28 37.5% (3)
Session 14: Top 1 frequent time interval	3–6 100% (1)	6–9 100% (1)	6–9 100% (2)	0–3 67% (2)	28–30 100% (2)	>30 100% (4)
Session 14: Top 2 frequent time interval	None	None	None	6–9 33% (1)	None	None

*(1) In Session 10 and 11, only three students were attending the class.*

**APPENDIX 9:** Choice of tasks in class at Stage 4: percentage (and Frequency) of active participation.

	Kelly	Amy	Andrew	Sam	Cora	Mike
Session 15: Top 1 choice	Vocabulary 64% (9)	Vocabulary 71% (5)	Vocabulary 83% (10)	Vocabulary 83% (5)	Vocabulary/Listening 40% (2)/40% (2)	Vocabulary/Listening 50% (2)/50% (2)
Session 15: Top 2 choice	Grammar/Listening 14% (2)/14% (2)	Grammar 29% (2)	Grammar 17% (2)	Listening 17% (1)	Grammar 20% (1)	None
Session 16: Top 1 choice	Vocabulary 62.5% (5)	Vocabulary/Grammar 50% (1)/50% (1)	Vocabulary 58% (7)	Vocabulary 67% (4)	Vocabulary/Grammar 50% (3)/50% (3)	Grammar 100% (2)
Session 16: Top 2 choice	Grammar 25% (2)	None	Grammar 25% (3)	Grammar 33% (2)	None	None
Session 17: Top 1 choice	Grammar 50% (4)	Vocabulary/Grammar 40% (2)/40% (2)	Vocabulary 80% (12)	Vocabulary 40% (4)	Vocabulary/Grammar 37.5% (3)/37.5% (3)	Listening 60% (3)
Session 17: Top 2 choice	Vocabulary 37.5% (3)	Listening 20% (1)	Grammar 20% (3)	Grammar/Listening 30% (3)/30% (3)	Listening 25% (2)	Vocabulary/Grammar 20% (1)/20% (1)
Session 18: Top 1 choice	Vocabulary 58% (7)	Vocabulary/Grammar 50% (1)/50% (1)	Vocabulary 64% (14)	Vocabulary 57% (4)	Vocabulary/Grammar 50% (3)/50% (3)	Vocabulary 67% (2)
Session 18: Top 2 choice	Grammar 42% (5)	None	Grammar 27% (6)	Grammar 43% (3)	None	Grammar 33% (1)
Session 19: Top 1 choice	Vocabulary 73% (11)	Absent	Absent	Absent	Vocabulary 50% (4)	Grammar 50% (4)
Session 19: Top 2 choice	Grammar 20% (3)	Absent	Absent	Absent	Grammar 37.5% (3)	Vocabulary/Listening 25% (2)/25% (2)

*(1) In Session 16 and 18, no listening tasks were included.*

**APPENDIX 10:** Persistence of task in class at Stage 4: percentage (and Frequency) of time intervals (between mistake and subsequent active participation) (time interval in minutes).

	Kelly	Amy	Andrew	Sam	Cora	Mike
Session 15: Top 1 frequent time interval	0–3 100% (1)	None	0–3 83% (5)	None	3–6 100% (2)	12-15 67% (2)
Session 15: Top 2 frequent time interval	None	None	0 17% (1)	None	None	9–12 33% (1)
Session 16: Top 1 frequent time interval	0–3 100% (1)	None	None	None	3–6 100% (2)	9–12 100% (2)
Session 16: Top 2 frequent time interval	None	None	None	None	None	None
Session 17: Top 1 frequent time interval	3–6 100% (1)	None	0–3 100% (2)	None	6–9 100% (2)	6–9 67% (2)
Session 17: Top 2 frequent time interval	None	None	None	None	None	9-12 33% (1)
Session 18: Top 1 frequent time interval	None	None	0-3 100% (3)	0-3 100% (1)	3–6/6–9 50% (1)	3–6 67% (2)
Session 18: Top 2 frequent time interval	None	None	None	None	None	6–9 33% (1)
Session 19: Top 1 frequent time interval	None	Absent	Absent	Absent	0–3 100% (2)	3–6 75% (3)
Session 19: Top 2 frequent time interval	None	Absent	Absent	Absent	None	0–3 25% (1)
